# Surgical Club of the South West, November Meeting 1989

**Published:** 1990-09

**Authors:** 


### Surgical Club of the South West

### Meeting in Bristol, November 1989

### BREAST SURGERY: A PERSONAL AUDIT FOR 1988; SOME ASPECTS OF HISTOLOGY AND CYTOLOGY

**A. John Webb**
Bristol Royal Infirmary

A personal series from 1988 was reviewed. The patients came from consulting practice with some problem cases from the Royal Infirmary. From 194 patients, 336 cytological procedures derived, including fine needle biopsies (F.N.A.B.C.).

Of the 29 carcinomas, 26 were primary lesions and there was one 'cancer' where cytology and histology remain in contention. Considering the 28 proven carcinomas, 7 were impalpable, mammographically revealed lesions. Cytological sensitivity (positive readings) was 85% and 100% (positive and suspicious readings). In the benign group of 131, were 58 patients with cystic dysplasia yielding a total of 156 aspirated cysts. Persistent cyst fluid atypia was detected in 1.3%, 2/1546 cysts, and in both instances occult carcinoma was discovered in the breasts. A total of 15 impalpable lesions (7 benign, 7 malignant, 1 problem) were successfully located, excised and radiologically proved. The ratio of benign to malignant operations was 31/27 (1/1).

Examples were illustrated and several points were emphasised.

(a) The importance of accurate detailed records and obsessive follow-up.

(b) The continuing value of the "triple assessment": clinical, mammography and F.N.A.B.C.

(c) The importance of always submitting cyst fluid to cytological examination.

(d) The challenge of early disease (D.C.I.S.) and unexpected pathology deriving from the Breast Screening Initiative (experiment).

(e) The recognition that managing breast problems can, at times, be very difficult.

### EVALUATION & SURGICAL CORRECTION OF OESOPHAGITIS AFTER PARTIAL GASTRECTOMY

**D. C. Gotley, D. E. Ball, R. W. Owen, R. C. N. Williamson & M. J. Cooper**
Bristol Royal Infirmary

Among 51 patients with refractory symptomatic reflux oesophagitis seen during an 18 month period, 8 (16%) had undergone previous partial gastrectomy. Either Billroth II (n=6) or Billroth I (n=2) resection had been carried out for peptic ulceration 18 months to 30 years beforehand. Each patient was evaluated by symptom scoring, endoscopy and 24-hour pH monitoring plus a 16-hour oesophageal aspiration study, in which 2-hourly aliquots were measured for acid, pepsin, conjugated and unconjugated bile acids and trypsin. Following a 45 cm Roux-en-Y gastroenterostomy, symptom scoring and endoscopy were repeated at 6 to 12 months in all 8 patients.

Pepsin, acid and unconjugated bile acids were infrequently found in oesophageal aspirates. Conjugated bile acids in concentrations of up to 30 mmol/1 and trypsin of up to 428 ug/ml were found in cases with severe oesophagitis, mostly during nocturnal rest. Oesophagitis, heartburn, regurgitation and bilious vomiting, present in all patients before operation, were eradicated by Roux-en-Y conversion. Chronic post-gastrectomy symptoms, such as early satiety, dumping, epigastric pain and diarrhoea, were not substantially improved by operation.

Post-gastrectomy oesophagitis in patients resistant to medical therapy seems likely to be caused by nocturnal exposure to trypsin aided by the presence of conjugated bile acids, and is controlled by a 45 cm Roux-en-Y conversion. This procedure is much less predictable in its effect on chronic "post-gastrectomy" symptoms.

### TRANSTHORACIC TRANSHIATAL OESOPHAGO GASTRIC RESECTION WITHOUT LAPAROTOMY FOR OESOPHAGEAL CARCINOMA

**K. Jeyasingham**
Frenchay Hospital, Bristol.

Thoracotomy for intrathoracic pathology in thoracic surgical units carries a mortality of well under 1% whilst transthoracic oesophageal resections for malignancy carry a mortality of 8.9% in thoracic surgical units of 25 to 32% in non-thoracic units. An analysis of morbidity leading to mortality after oesophageal resections showed that nearly 60% were due to bronchopneumonia. Factors such as underlying chronic lung disease, ischaemic heart and circulatory disease, contribute to a higher mortality in the elderly (>70 years of age). Attempts at improving the outcome have included high dependency postoperative care in specialised centres, epidural analgesia, ventilatory support, prophylactic low dose Heparin and antibiotics, and modifications to surgical technique.

Transhiatal blunt oesophagectomy without thoracotomy was resurrected from the long past with the hope of reducing morbidity and mortality. This has, however, not proved to be the case in non-specialised units. Transhiatal oesophago gastric mobilisation and resection via a right thoracotomy for mid oesophageal carcinoma has been described as a feasible procedure. The present author has practised a transhiatal mobilisation of the stomach via a left thoracotomy for resection of lower oesophageal carcinoma and undilatable benign strictures in the over 70 year olds. The mortality for resection of benign undilatable strictures in 34 patients over the age of 70 years was 3, whilst that for 19 patients over 70 years of age with lower oeosphageal carcinoma was 3. In a three year follow-up of 12 such operated patients with carcinoma, the survival was 7 whilst of 9 patients available for a five year follow-up, 5 patients have survived. Thus, a transthoracic transhiatal oesophago-gastric resection without laparotomy yields a relatively low mortality in the over 70 years of age without compromising their survival chances.

### SURGERY OF THE ASCENDING AORTA

**J. D. Wisheart**
Bristol Royal Infirmary

Between 1975 and 1988, 45 operations were performed to replace the ascending aorta. The pathology was dissection in 28, annuloaortic ectasia in 11, Marfan's Syndrome in 5 and 1 other. Twenty emergency operations were performed, of which 187 were for acute dissection. Aortic replacement was supracoronary in 23, with or without aortic valve replacement, while composite replacement of the aorta and aortic valve with re-implantation of the coronary arteries was performed in 22. Thirty-day mortality was 16% for the whole group: emergency operations 25%, elective 8%; for dissection 18%, annulo-aortic ectasia 9%, Marfan's Syndrome 0% Late Survival of those who left hospital was 86%, and 75% at 5 and 7 years respectively. The causes of early and late death, and late morbidity, were mainly related to the aortic pathology and underline the importance of recognising that the operation is just one event in the management of the patient.

### SURGERY OF ACUTE PANCREATITIS

**D. Alderson**
Bristol Royal Infirmary

Acute pancreatitis continues to have a mortality of about 10%. Early deaths are usually due to renal failure, myocardial events of respiratory insufficiency. Late deaths characteristically occur beyond the second week of the illness and relate to the development of infection in necrotic pancreas and peri-pancreatic tissues. A study has been set up to identify those patients with necrosis, detect secondary infection and thus define a group where surgery seems appropriate, since the mortality of conservatively managed infected necrosis approaches 100%. Of 38 patients with acute pancreatitis, 17 have had "severe" attacks based on biochemical (Glasgow) criteria and/or serum C-reactive protein levels >120 mg/l. These patients have then undergone dynamic C.T. scanning of the pancreas ± percutaneous aspiration of any necrotic areas performed 7–10 days after the onset of the illness. Surgery has followed in 8 patients (4 for infection, 4 for pseudocyst or pancreatitis). There have been no deaths in the group of patients with "mild" attacks managed conservatively. There have been 2 deaths in the "severe" group—one due to the multi-system failure in the first week of the illness, and one after surgery. This represents an overall mortality of 5.3%, and an operative mortality of 12.5%. Recognition and appropriate treatment of necrosis can lead to a reduced mortality from acute pancreatitis.

### REVIEW OF EXTRACORPOREAL SHOCK WAVE LITHOTRIPSY FOR UPPER URINARY TRACT STONES September 1988—August 1989

**M. Wills**
Southmead Hospital, Bristol

Extracorporal Shock Wave Lithotripsy (ESWL) has been available at Southmead Hospital, Bristol for the treatment of Upper Urinary Tract Stones since April 1988. This is the result of a generous donation by Mr John James who provided funds to purchase a Siemens Lithostar, a second generation Lithotriptor.

During the year September 1988 to August 1989 634 patients were given a total of 982 treatments, an average of 1.54 treatments per patient. Of these 372 patients (58%) were referred from Hospitals within the South West Region Health Authority. Patients were also treated from Wales, West Midlands, Wessex and Oxford. The youngest patient to be treated was aged 22 months and the oldest 88 years. 57% of patients were treated as day case admissions.

The stone types treated were as follows: Pelvis 188 patients, Calyceal 260 patients, Staghorn 155 patients, Ureteric 115 patients. Some patients had stones in more than one category. Over 100 patients were treated with Ureteric Stones, this being possible with the Lithostar because of the facility for x-ray screening.

The average time for treatment was 1 hour. 10% of patients underwent Lithotripsy under general anaesthesia, the majority for insertion of a double J ureteric sent to aid the passage of fragments resulting from stones larger than 2 cms in diameter. 64 patients had a DJ Stent *in situ* and 15 patients had a nephrostomy *in situ* at referral.

Five patients with spina bifida, 2 patients with horseshoe kidney and 2 patients with stones in a transplanted kidney were treated. Complications were minimal during treatment.

The overall results produced a fragmentation rate of 80% and a stone free rate of 76%. For small stones the stone free rate was 82% but for Staghorn Calculi this fell to 65%.

ESWL provides a non invasive treatment for upper urinary tract stones with minial complications.

### COLECTOMY AND ILEORECTAL ANASTOMOSIS IMPROVES SENSORY ANORECTAL AWARENESS AND RECTAL EMPTYING IN SLOW TRANSIT CONSTIPATION

**G. S. Duthie, D. C. C. Bartolo**
Bristol Royal Infirmary

Slow transit constipation (STC) can be associated with anismus (STCA) [spastic pelvic floor function] or with normal pelvic floor function (STCN). Both groups may be successfully treated by colectomy and ileorectal anastomosis (IRA). We have investigated the physiological changes by mucosal electrosensitivity, volume to first rectal awareness, and radiology, in 102 patients with slow transit constipation. 54% were radiologically defined as STCA, 46% STCN. Both groups have impaired sensation (STCA: 6.9, 7.0, 8.4 mAmps at 1, 2 and 3 cm respectively; STCN 8.6, 9.1, 10.9; vs controls 4.6, 4.3, 5.4: $p<0.001^*$) and STCA had a high volume requirement for rectal awareness (STCA 162.0 ml vs STCN 77.3: $p=0.008$).

Thirty two patients underwent IRA (STCA 47%, STCN 53%) and postoperative sensation improved in the upper anal canal in both groups (STCA 17.5 mAmps preop vs 12 postop; STCN 20 vs 13; $p<0.02$;) as did rectal awareness STCA 70 ml vs 45; STCN 95 vs 50: $p<0.05^+$). In addition postoperative defaecography showed significant resolution of anismus and the ability to empty the rectum [$x^2=5.333$; $0.05>p>0.01$).

Thus improved postoperative function is related to improved sensory awareness in the anorectum and this may be implicated in the resolution of anismus.

* Mann Whitney U test
\+ Wilcoxon signed rank paired test

### PARATHYROID TRANSPLANTATION

**J. R. Farndon**
Bristol Royal Infirmary

The clinical impetus to develop parathyroid transplantation came from the observed high incidence of hypoparathyroidism following thyroidectomy in patients treated at the turn of the century. Halsted carried out successful experimental transplantation in dogs.

The indication for parathyroid transplantation is diffuse four-gland hyperplasia—primary or secondary. Secondary hyperparathyroidism due to chronic renal failure is by far the commonest indication. Frozen section histology allows the identification of the most normal gland and 12–15 fragments [1 × 1 × 3 mm) from this are implanted into separate pockets in the brachioadialis muscle of the forearm. Comparison of PTH concentrations from ante cubital veins from the grafted arm can be compared with concentrations from the non-grafted side—gradients. These confirm graft function and can be a marker for recurrent graft dependent disease. If confirmed—resection of half the fragments under local anaesthetic usually allows restoration of normal parathyroid function. Forearm grafting obviates the need for neck re-exploration.

In secondary disease reversal of radiographic and histological features of renal osteodystrophy will occur in 70% of patients. Bone pain may persist. Small vessel calcification can be reduced but not that in medium or large arteries. Pruritis will only resolve fully following renal transplantation. Myopathy is improved following parathyroidectomy.

A rare complication of the procedure is parathyromatosis—that propensity of parathyroid adenoma cells to seed and multiply abnormally in ectopic sites. The presence of an ademona or adenomatous hyperplasia precludes the use of such tissue for transplantation.

### RUPTURED MYCOTIC ANEURYSM DUE TO SALMONELLA

**A. R. Baker**
Frenchay Hospital, Bristol

A 55 year old man was admitted as an emergency eleven days after returning from a holiday in Madeira. He presented with a one week history of diarrhoea and rigors together with a twelve hour history of back pain. Blood cultures grew Salmonella virchow. Three days after admission he developed a scrotal haematoma and two days later collapsed with severe abdominal pain, hypotension and a large pulsatile abdominal mass.

Emergency laparotomy confirmed the diagnosis of a ruptured mycotic aortic aneurysm. The aorta was closed proximal and distal to the sac and the legs revascularised with an axiallo-bifemoral bypass graft. The infection was treated with prolonged intravenous ciprofloxacin and the patient recovered gradually over several weeks. Salmonella virchow was grown from the aortic wall.

Salmonella infected aneurysms are rare and carry a high mortality. Before 1969 only one survivor is recorded. Up to 1987 the mortality was 56%. Mycotic aneurysms probably arise by bacteria colonising atheromatous lesions during episodes of bacteraemia. The diagnosis should be considered in any patient with bacteraemia who develops pain in the back, abdomen or chest. The risk of endothelial infection in patients over 50 years old with salmonella has been estimated at 25%. It is therefore advisable for patients over this age contracting a salmonella infection to be treated with antibiotics.

### ELECTRONIC DETECTION OF GLOVE PUNCTURES DURING SURGERY

**A. J. Hamer**
Bristol Royal Infirmary

Surgical gloves puncture with alarming regularity. It is disturbing that in 50% of cases the surgeon may be unaware that a perforation has occurred (1).

In the light of the ever increasing number of HIV and Hepatitis B positive patients, it would be most desirable to be made aware of a glove puncture as soon as it occurred, so that gloves could be changed immediately, preventing prolonged contact of patients' body fluid with surgeons' skin. Likewise, cotton gowns and drapes are not impervious to organisms, particularly when they are wet.

An electronic device intended to detect direct cóntact of patients' body fluids with surgeons' skin has been described, where a breakdown in the normal very considerable electrical resistance between surgeon and patient, as a result of direct fluid contact between the two, causes an audible alarm tO sound (2). I am able to report preliminary experience of its use in 51 cases.

The alarm sounded 41 times in 17 cases, the maximum number per case being 5. Glove punctures (determined by inflation under water) were responsible on 11 occasions (5 when double gloved i.e. punctures through both pairs), and damp gown sleeves touching the wound caused 29 alarms. The device alarmed only once without explanation.

1. BROUGH, S. J., HUNT, T. M., BARRIE, W. W. (1988) Surgical glove performations. *Br. J. Surg.* **75**, 317.
2. HAMER, A. J. (1987) Electronic device for the detection of breaches in asepsis during surgical procedures. *Br. J. Surg.* **74**, 1038–1039.

### RECTOPEXY FOR RECTAL PROLAPSE WITH INCONTINENCE

**G. S. Duthie, D. C. C. Bartolo and P. Lewis**
Bristol Royal Infirmary

Rectal prolapse is commonly complicated by faecal incontinence. Abdominal rectopexy will control the prolapse and often improves continence. We have investigated 57 patients (8 male: 49 female; ages 62.5 (23–83)); 20 resection rectopexy (MR), 9 Ivalon rectopexy (IR), 8 suture rectopexy (SR) to determine if this is due to post operative constipation, improved sphincter function, rectal morphological changes or improved sensation.

Ages, symptoms, and follow-up are all similar. Prolapse was controlled in all cases. Continence (to solid and liquid stool) was significantly improved (RR 27% continent pre-op vs 91% post-op; MR 27% vs 73%; IR 40% vs 80%; SR 25% vs 87%: $P<0.05^{*}$). Stool frequency, straining at stool and incomplete emptying were unchanged postoperatively. Sphincter length and manometry were similarly unchanged. Anorectal sensation was improved in the rectum (RR first sensitivty pre-op 15 mAmp vs 13 post-op; MR 18 vs 13; IR 15 vs 11.2; SR 25.5 vs 13: $P<0.05^{+}$). Changes in anorectal angulation did not correlate with improved continence. In conclusion; post operative constipation and improved sphincter function are not responsible for restoration of continence, but improved anorectal sensation may have a significant role to play.

* Chi-squared analysis
+ Wilcoxon signed ranks paired test

### FATAL FATTY LIVER

**Dhafir Al-Okati, Charles Nankivell and Nassif B. N. Ibrahim**
Departments of Histopathology and General Surgery, Frenchay Hospital, Bristol

A 36-year-old woman, a known alcoholic and epileptic, was brought to hospital with a 12 hour history of abdominal pain. On arrival she was unconscious with unrecordable blood pressure. After fluid resuscitation she became semi-conscious. Abdominal examination revealed a tender epigastrium. An urgent laparotomy revealed an enormous pale yellow liver and also mild pancreatitis. Results of blood tests taken immediately pre-operatively, became available later, showed blood glucose level of less than 1 mmol/L, raised amylase, normal ALT, slightly raised alkaline phosphatase, low albumin and low total protein. Bilirubin was at the upper limit of normal. Blood alcohol was 78 mg/L. Despite full supportive therapy, she rapidly developed progressive liver, renal and respiratory failure. She sustained a cardiac arrest and died forty hours following admission.

At post mortem examination the only significant finding was the markedly enlarged fatty liver (weight 3000 grams). Histology confirmed severe hepatic fatty change with no significant fibrosis or inflammatory cellular infiltration. Sections from all other major organs revealed no significant abnormality.

This is a case of fatty liver—related sudden death, an entity in need of a much wider recognition. Two large series from the United States (a total of 522 cases of fatty liver—related death) emphasised the negative or very low blood alcohol levels in over 60% of cases, a finding consistent with several theories linking fatty liver—related deaths to some form of acute or hyperacute ethanol withdrawal phenomenon. The majority of these cases occured in the 25–44 year old age group. Autopsies revealed enlarged fatty livers with no other significant pathological findings. On histology, the liver shows no cirrhosis, no significant fibrosis and in over 80% of cases there is no significant inflammatory cellular infiltration. Proposed mechanisms of fatty liver death included hypoglycaemia, fat embolism (although there is strong evidence suggestive that pulmonary fat embolism is not the cause of death in the majority of cases), hypomagnesemia, general ethanol withdrawal syndrome and ethanol-induced neurotransmitter changes.

